# Data-driven subtyping of Parkinson’s disease: comparison of current methodologies and application to the Bochum PNS cohort

**DOI:** 10.1007/s00702-023-02627-4

**Published:** 2023-03-31

**Authors:** Qiang Chen, Raphael Scherbaum, Ralf Gold, Kalliopi Pitarokoili, Axel Mosig, Samis Zella, Lars Tönges

**Affiliations:** 1 Department of Neurology, St. Josef-Hospital, Ruhr University Bochum, 44791 Bochum, Germany; 2 Department of Psychiatry, Landschaftsverband Rheinland-Klinik (LVR-Klinik), 40764 Langenfeld, Germany; 3 Medizinisches Zentrum für Erwachsene mit Behinderung (MZEB), Landschaftsverband Rheinland-Klinik, 40764 Langenfeld, Germany; 4 Center for Protein Diagnostics (ProDi), Ruhr University Bochum, 44801 Bochum, Germany; 5 Immune-Mediated Neuropathies Biobank (INHIBIT), Ruhr-University Bochum, Bochum, Germany; 6 Bioinformatics Group, Ruhr University Bochum, 44801 Bochum, Germany

**Keywords:** Parkinson’s disease, Data-driven analysis, Subtype, Machine learning, Clustering

## Abstract

Considerable efforts have been made to better describe and identify Parkinson's disease (PD) subtypes. Cluster analyses have been proposed as an unbiased development approach for PD subtypes that could facilitate their identification, tracking of progression, and evaluation of therapeutic responses. A data-driven clustering analysis was applied to a PD cohort of 114 subjects enrolled at St. Josef-Hospital of the Ruhr University in Bochum (Germany). A wide spectrum of motor and non-motor scores including polyneuropathy-related measures was included into the analysis. *K*-means and hierarchical agglomerative clustering were performed to identify PD subtypes. Silhouette and Calinski–Harabasz Score Elbow were then employed as supporting evaluation metrics for determining the optimal number of clusters. Principal Component Analysis (PCA), analysis of variance (ANOVA), and analysis of covariance (ANCOVA) were conducted to determine the relevance of each score for the clusters’ definition. Three PD cluster subtypes were identified: early onset mild type, intermediate type, and late-onset severe type. The between-cluster analysis consistently showed highly significant differences (*P* < 0.01), except for one of the scores measuring polyneuropathy (Neuropathy Disability Score; *P* = 0.609) and Levodopa dosage (*P* = 0.226). Parkinson’s Disease Questionnaire (PDQ-39), Non-motor Symptom Questionnaire (NMSQuest), and the MDS-UPDRS Part II were found to be crucial factors for PD subtype differentiation. The present analysis identifies a specific set of criteria for PD subtyping based on an extensive panel of clinical and paraclinical scores. This analysis provides a foundation for further development of PD subtyping, including *k*-means and hierarchical agglomerative clustering.

*Trial registration*: DRKS00020752, February 7, 2020, retrospectively registered.

**Supplementary Information:**

The online version contains supplementary material available at 10.1007/s00702-023-02627-4.

## Introduction

Parkinson's disease (PD) is a complex and heterogeneous disorder in both clinical and paraclinical terms. The clinical presentation and progression of PD are highly variable and present different scenarios not only in various stages of the disease but also within a single stage (Barone et al. 2009; Beiske et al. 2009; Defazio et al. 2008; Fereshtehnejad et al. 2017; Ford 1998; Gallagher et al. 2010; Goetz et al. 1986; Hendricks and Khasawneh 2021; Jankovic et al. 1990; Kalia and Lang 2015; Koller 1984; Lawton et al. 2018; Snider et al. 1976; Wasner and Deuschl 2012). The identification of PD subtypes may therefore lead to further insights into pathophysiological mechanisms of disease but could also identify novel therapeutic targets and ultimately lead to improvements in patient care.

Usually, the clinical presentation of PD is described with three main clinical phenotypes: tremor-dominant phenotype, akinetic-rigid phenotype, and mixed or equivalence phenotype (the latter being a combination of the other two phenotypes without any dominant symptoms (The German Neurological Society 2016). The course of disease has been shown to be associated to the clinical phenotype as with the tremor-dominant phenotype developing more slowly with a less severe course than the akinetic-rigid or equivalence phenotype (Jankovic et al. 1990; Wojtala et al. 2019).

However, the depth of phenotypic information in the aforementioned studies was often variable and limited. Each of these subgroups shows different clinical progression, and disease symptomatology does not only consist of motor symptoms but also of disabilities from non-motor deficits (e.g., depression, anxiety, fatigue, orthostatic hypotension, sleep disturbances, polyneuropathic hypoesthesia, and thus movement difficulties). So far, relatively few studies have focused on a more thorough analysis of these complex manifestations of the disease (Mestre et al. 2021). These are characterized by high variability not only between patients or within the same patient, but also by high variability depending on the clinical disease stage (Barone et al. 2009; Beiske et al. 2009; Defazio et al. 2008; Ford 1998; Gallagher et al. 2010; Goetz et al. 1986; Jankowicz et al. 1986; Kalia and Lang 2015; Koller 1984; Nègre-Pagès et al. 2008; Snider et al. 1976; Wasner and Deuschl 2012). Interestingly, previous work showed that polyneuropathy (PNP) has a high prevalence in people with PD and can be associated with non-motor and motor symptoms of PD, as well as with the disease severity (Kühn et al. 2020). PNP as associated disease criterion has never been applied before in disease pattern clustering analyses.

In this paper, the authors propose a data-driven subtyping method that integrates motor and non-motor characteristics, including polyneuropathy-related scores of PD patients.

The main objectives of the present study were: (1) to perform cluster analysis using machine learning algorithm; (2) to identify the PD subtypes and compare the clinical characteristics of each subtype; and (3) to identify the crucial factors to differentiate the PD subtypes and analyze the association between those factors.

## Data and methods

This original paper adhered to the STROBE (Strengthening the Reporting of Observational Studies in Epidemiology) guidelines for reporting observational studies (Elm et al. 2007). By following these guidelines, the authors aimed to ensure the transparency, completeness, and rigor of the current study, and to facilitate the critical appraisal and interpretation of the present findings by readers and reviewers.

### Study design

The study was performed as a data-driven subtyping approach based on a cross-sectional sample from a single-center prospective observational cohort study (Kühn et al. 2020). The study was approved by the Institutional Review Board of the Medical Faculty of the Ruhr University Bochum on September 12, 2018 (Register No. 18-6360), registered in the German Clinical Trials Register (DRKS-ID: DRKS00020752), and conducted in accordance with the ethical standards of the Declaration of Helsinki.

### Setting and participants

Data for the present analysis were collected from October 2018 to January 2022 at the department of neurology of a university medical center (St. Josef-Hospital of Ruhr University Bochum, Germany). As published previously (Kühn et al. 2020), eligibility criteria comprised an age over 18 years, a diagnosis consistent with the PD diagnostic criteria according to both the United Kingdom Parkinson’s Society Brain Bank criteria (Gibb and Lees 1988) and the Movement Disorders Society’s Criteria for Parkinson’s disease (Postuma et al. 2015), and written informed consent. Exclusion criteria comprised causes of neuropathy, such as diagnoses of diabetes mellitus or alcohol dependence disorder, as well as severe dementia, insufficient language skills, illiteracy, and acute mental disorders. Inpatients and outpatients of the department were screened for eligibility during the period of recruitment by review of the hospital information system. The participants included in this analysis were or are followed up over several years, but only data from the baseline visit were included into the present analysis.

### Variables and data sources

A comprehensive assessment including clinical examination, and patient-report questionnaires were performed. For the cluster analysis, 14 features were included:

- Demographic information: age and disease duration since diagnosis of PD.
- Motor symptoms: Movement Disorder Society-Unified Parkinson’s Disease Rating Scale (MDS-UPDRS) Part II (motor experiences of daily living), Part III (motor examination), and Part IV (motor complications); The Hoehn and Yahr (H&Y) Stage.
- Non-motor symptoms: MDS-UPDRS Part I (non-motor experiences of daily living), Non-motor Symptom Questionnaire (NMSQuest) (Chaudhuri et al. 2006), Scales for Outcomes in Parkinson’s Disease-Autonomic (SCOPA-AUT) (Visser et al. 2004), and Parkinson’s Disease Questionnaire (PDQ-39) (Jenkinson et al. 1997).
- Cognitive function: Montreal Cognitive Assessment (MoCA) (Nasreddine et al. 2005).
- Other feature: levodopa equivalent daily dose (LED).
- Polyneuropathy examination: modified Neuropathy Disability Score (NDS) (Dyck et al. 1980; Xiong et al. 2015) and modified Neuropathy Symptom Score (NSS) (Dyck et al. 1980).

### Statistical methods

A total of 114 patients were included in the present study, and the missing values (4.45%) of the 14 features were imputed using mean values for the entire data, which are presented as means with standard deviations. Data were tested for normality and homogeneity of variance with Quantile–quantile plot, Shapiro–Wilk test, and Levene test, respectively. Univariate statistical tests were performed with Pearson's chi-square tests for categorical data. For continuous variables, analysis of variance (ANOVA) with Kruskal–Wallis *H* test and post hoc analysis with Dunn’s test with Holm adjustment were performed. Analysis of covariance (ANCOVA) was conducted to adjust between-clusters comparisons for age and disease duration as potential covariates. Correlations between the 14 parameters were calculated with Spearman's rank method. Global significance was set at *α* < 0.05. Statistical analyses were performed with Python version 3.9.14. Due to the observational and exploratory nature of the study, no sample size calculation was performed.

### Choice of appropriate clustering methodology

Clustering is a technique used in data analysis to group similar data points together. Two of the most widely used methods for clustering are hierarchical and partitioning (Sharma 1996; Fraley and Raftery 1998). The hierarchical method is a non-partitioning approach. The clusters are represented hierarchically through a dendrogram. A dendrogram is a tree-like structure where the leaves represent individual data points, and the branches represent groups of data points that are similar to one another. Depending upon whether this hierarchical representation is created in top–down or bottom–up fashion, these representations may be considered either agglomerative or divisive (Aggarwal and Reddy 2013). Hierarchical method is more structured, and it is easier to decide the number of clusters. However, the resulting clustering is time complexity and may be sensitive to the ordering by which the data are presented. Furthermore, hierarchical clustering technique is very subtle for outlier.

In contrast, partitioning clustering involves dividing the data points into a fixed number of clusters, typically using algorithms like *k*-means. *K*-means clustering is an unsupervised machine learning algorithm; a cluster is represented by its centroid, which is usually the mean of points within a cluster. It works by minimizing the sum-of-squares distance of the data points in the same cluster. The *k*-means method is considered one of the simplest and most classical methods for data clustering (Jain 2010). It is also the most widely used methods in cluster analysis (Aggarwal and Reddy 2013). *K*-means method produces tighter clusters than hierarchical method and runs faster if the variables are large (Pandya and Saket 2020). A disadvantage is that the number of clustered must be specified.

Given the advantages and drawbacks that have been previously discussed, the present study employed a combination of both *k*-means and hierarchical method to cluster the data. The primary method used was *k*-means, while the hierarchical method was utilized as a validation tool to confirm the robustness of the cluster number obtained from the primary method. *K*-means++ (Arthur and Vassilvitskii 2007) is an advanced version of standard *k*-means algorithm that improves the way of selecting initial centroids. Instead of choosing them randomly, *k*-means++ selects a data point farther away from any existing centroid with probability proportional to the squared distance to the closest existing centroid, which leads to better performance and faster convergence. A previous review of PD cluster analysis studies conducted between 1999 and 2021 identified that the *k*-means cluster method was the most used approach, being utilized in 13 out of 24 studies. The hierarchical method, on the other hand, was employed in three studies (Hendricks and Khasawneh 2021). The 14 features included in the current clustering analysis are all numerical variables, and *k*-means is a popular distance-based clustering algorithm that is particularly well-suited to numerical data, as it uses the Euclidean distance to measure the similarity between data points. As *k*-means algorithm is sensitive to the scale of variables, to ensure accurate results, the data were first standardized and transformed. Silhouette method and Calinski–Harabasz scores were applied to determine the optimal number of clusters. To validate the optimum solution of *K*, hierarchical clustering was implemented as a final step before applying the *k*-means++ algorithm.

### Cluster analysis methods

The *k*-means++ algorithm, hierarchical clustering, and Principal Component Analysis (PCA) were performed using scikit-learn (Pedregosa et al. 2011). Correlation circle and Biplot were generated using the FactoMineR and Factoextra packages (Lê et al. 2008) in R programming language (version 4.2.0) (R Core Team 2021).

## Results

The total analysis set included 114 participants and consisted of 66 (57.9%) males and 48 (42.1%) females. In reference to the selected 14 features, the following presents a summary of the descriptive statistical results (mean ± SD):

- Demographic information: age (70.49 ± 10.02) and disease duration (8.14 ± 5.10).
- Motor symptoms: MDS-UPDRS Part II (13.41 ± 9.41), Part III (30.15 ± 14.59), and Part IV (4.01 ± 3.95); H&Y (2.65 ± 0.77).
- Non-motor symptoms: MDS-UPDRS Part I (11.91 ± 6.26), NMSQuest (9.76 ± 5.20), SCOPA-AUT (14.00 ± 7.94), and PDQ-39 (42.70 ± 27.82).
- Cognitive function: MoCA (22.60 ± 4.00).
- Other feature: levodopa equivalent daily dose (657.97 ± 411.55).
- Polyneuropathy examination: NDS (3.69 ± 2.59) and NSS (4.88 ± 3.01).

Ensuring the validity of the results obtained from a PD analysis requires the identification and adjustment of potential confounders (Hubble et al. 2015). Aging can affect the movement system independently of PD, and advanced age has previously been proposed to be associated with a more severe PD phenotype with accelerated progression (Raket et al. 2022). In the context of Parkinson's disease research, age and disease duration are considered as confounders as they have the potential to influence the outcome of the study. To accurately compare results between clusters, ANCOVA (analysis of covariance) is employed as a statistical technique to control for the effect of these confounders. Furthermore, to bolster the robustness of the analysis, bootstrapping is utilized to estimate the 95% Confidence Interval (CI) of the adjusted means, thereby providing a more comprehensive examination of the precision of the between-cluster comparison.

### Determination of optimal number of clusters

The number of clusters (*K*) is a crucial parameter in the *k*-means algorithm and must be set prior to running the algorithm. The *k*-means algorithm assumes that the data can be divided into a fixed number of clusters, and this number is defined by the *K* parameter. The algorithm operates by defining a fixed number of centroids, or cluster centers, and then iteratively assigning data points to the cluster with the closest centroid. The centroids are subsequently updated according to the mean of the data points assigned to each cluster. Without an accurate and appropriate value of *K*, the *k*-means algorithm may not be able to partition the data into meaningful clusters.

**Fig. 1:**
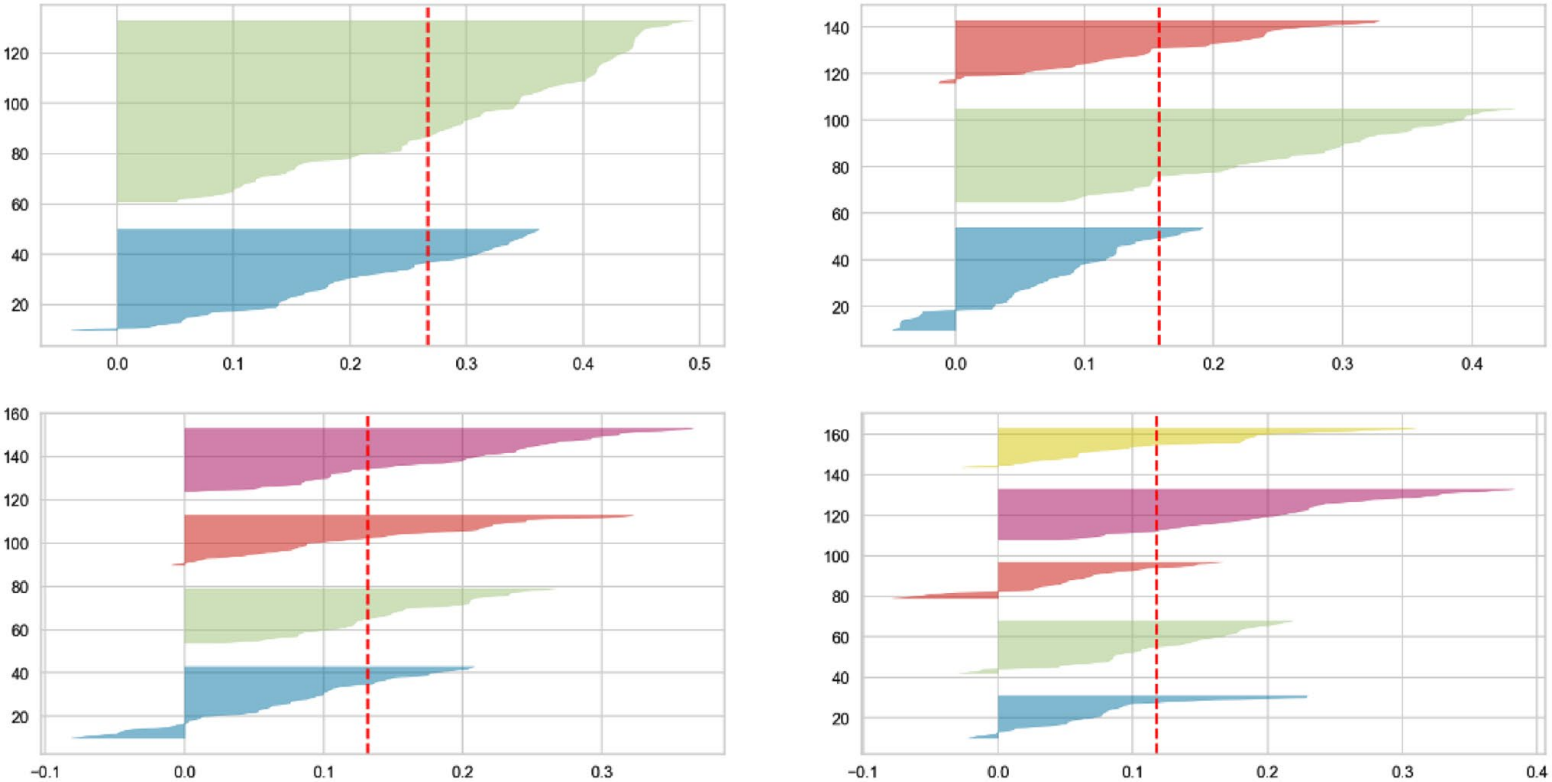
The silhouette analysis of *k*-means clustering (number of clusters = 2, 3, 4 and 5)

Two methods were used to specify the optimal number of clusters (*K*). The first one is the silhouette method. The silhouette coefficient, which ranges between − 1 and 1, was calculated and it indicates how similar a data point is within-cluster compared to other clusters. The two-cluster solution and the three-cluster solution had the highest average silhouette coefficient (0.243 and 0.158, respectively). A silhouette analysis of *k*-means clustering with different numbers of clusters was performed (Fig. 1). The two-cluster option formed one cluster consisting of 74 patients and the other one of 40 patients. In comparison, the three-cluster solution formed clusters with 49, 40, and 25 patients, respectively.

**Fig. 2:**
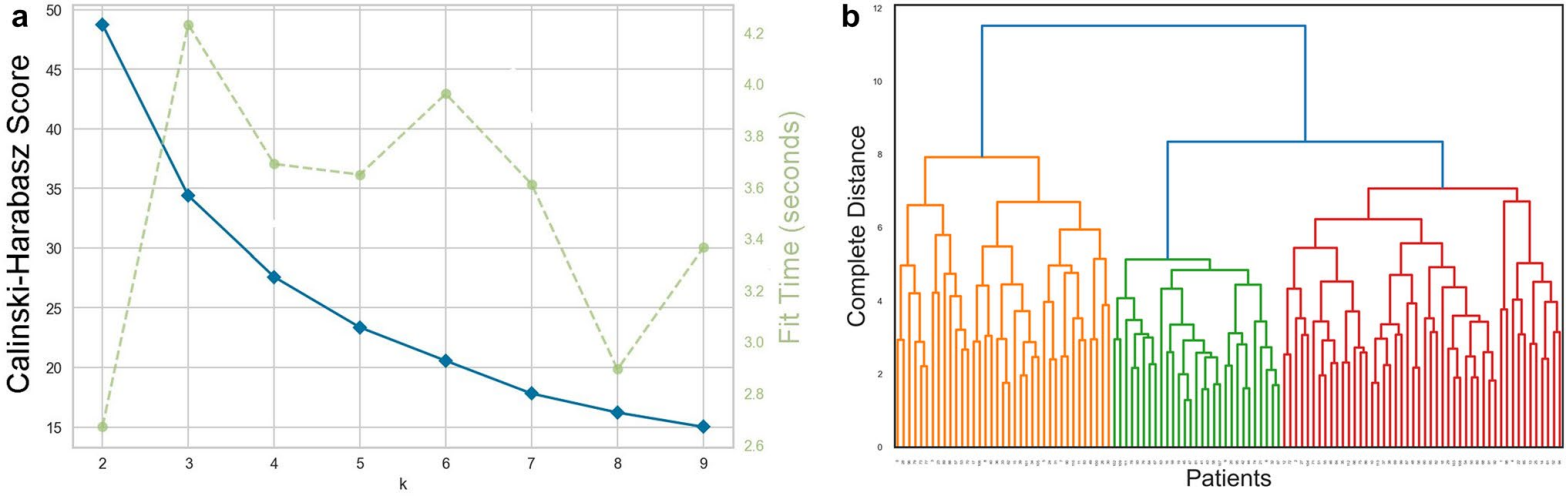
**a** The Calinski–Harabasz Score Elbow: the potential numbers of clusters are those values of *k* for which the angles are formed (blue line). There is elbow for *K* = 3; the optimal number of clusters corresponds to the solution with the highest Calinski–Harabasz Score (green dotted line); for *K* = 3, there is a highest value of Calinski–Harabasz Score. **b** The dendrogram of hierarchical clustering using complete distance, a well-distinguished clusters of three can be observed

The second method is the Calinski–Harabasz Score Elbow (Fig. 2a). It suggests that the optimal number of clusters is three, which has the highest Calinski and Harabasz score. The potential numbers of clusters are those values of *K* for which the angles are formed; an elbow can also be observed for *K* = 3.

Additionally, hierarchical clustering was performed and the resulting dendrogram (Fig. 2b) revealed a well-balanced cluster of three groups.

Both *k*-means clustering and hierarchical clustering suggested three as an optimal number of clusters (Fig. 2a, b). The silhouette and Calinski–Harabasz Score supported the optimal number of clusters issued from the *k*-means clustering. The authors identified the three-cluster solution as optimal, because it provided a better-balanced data distribution and clinical relevance than the two-cluster solution.

### Principal component analysis (PCA)

**Fig. 3:**
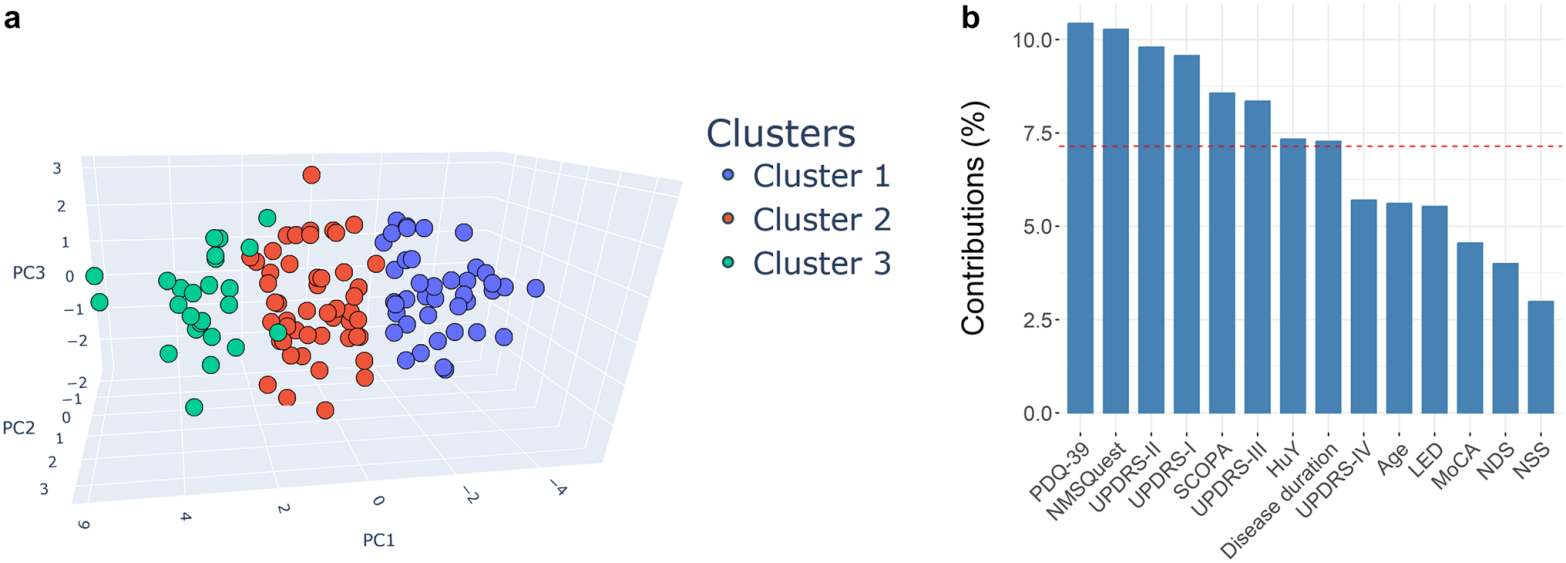
**a** The three-dimensional PCA (PC1, PC2, and PC3) visualization of the three clusters formed by the *k*-means clustering algorithm. **b** Contribution of features to both component 1 and 2 in the PCA, and the contributions of features are expressed in percentage; if the contribution of the features were uniform, the expected average contribution would be 7.14% (1/length (features) = 1/14 = 7.14%), which is indicated with the red dashed line on the graph. For the component 1 and 2, a feature with a contribution larger than this cut-off could be considered as important feature, the three most important features are therefore: PQD-39, NMSQuest, and MDS-UPDRS Part II

**Fig. 4:**
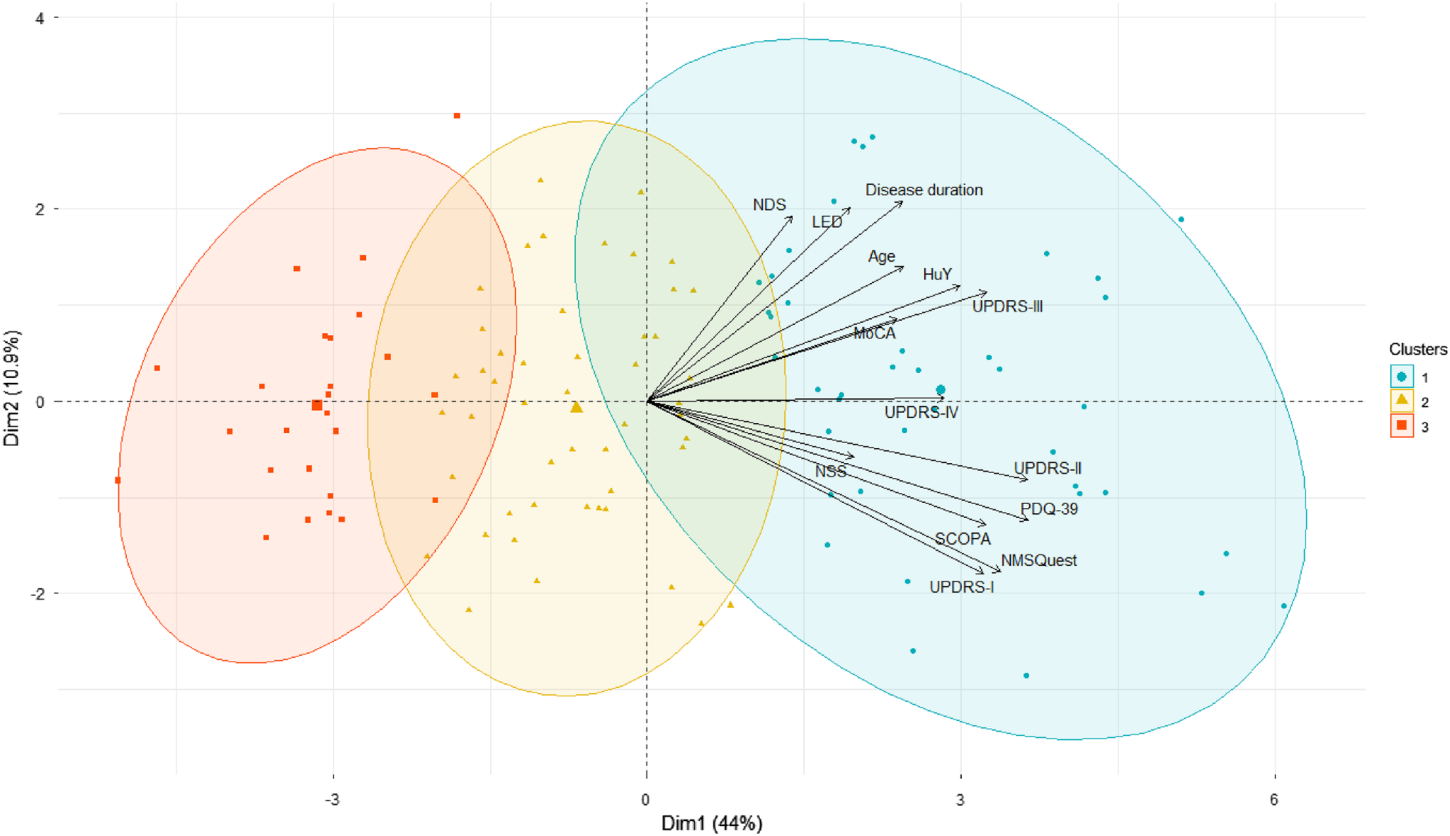
Biplot visualization of the clustering: the plot shows patients’ data as points in the plane, which is formed by two principal components as axes with the explained variances, respectively (Dim1 = 44% and Dim2 = 10.9%). The length of the features and in particular its angle with the principal component axis shows the degree of its contribution to that principal component. The angles between the feature vectors show their correlation: small angles represent high positive correlation, which can be observed such as between NMS and UPDRS-1 or between PDQ-39 and UPDRS II, etc.; right angles represent lack of correlation, such as between UPDRS I and NDS. As MoCA values and the other 13 variables are measured in opposite directions, with higher MoCA values indicating positive cognitive conditions and higher values for the other 13 variables corresponding to worse disease manifestations. To present the subtypes in a consistent manner, the original MoCA values were replaced by the new values obtained by subtracting the original data from the maximal MoCA value of 30

To reduce the high dimensionality of the features to three dimensions, the Principal Component Analysis (PCA) was performed, allowing the visualization of the resulting *k*-mean clusters (Fig. 3a). To analyze how important each feature was for the characters of the different clusters, the loading scores for the first and second principal components were calculated (Fig. S1). The loading score represents the importance of each feature in defining the subtypes. In terms of the first principal component (explained variance ratio 43.9%), PDQ-39 and MDS-UPDRS Part II had the largest loading scores (0.342 and 0.341, respectively) and therefore contribute mostly to the first principal component, followed by NMS (0.317), MDS-UPDRS Part III (0.305), SCOPA-AUT (0.304) and MDS-UPDRS Part I (0.302). For the second principal component (explained variance ratio 10.9%), the disease duration and LED were the most important features with a loading score of 0.393 and 0.380, respectively. For both principal components, the contribution of variables was calculated and the top three most important features in characterizing the subtypes were PDQ-39, NMSQuest, and MDS-UPDRS Part II (Fig. 3b). Furthermore, a biplot of the three clusters with the PCA and the loadings of the 14 features is illustrated in Fig. 4.

### Subtype identification

**Fig. 5:**
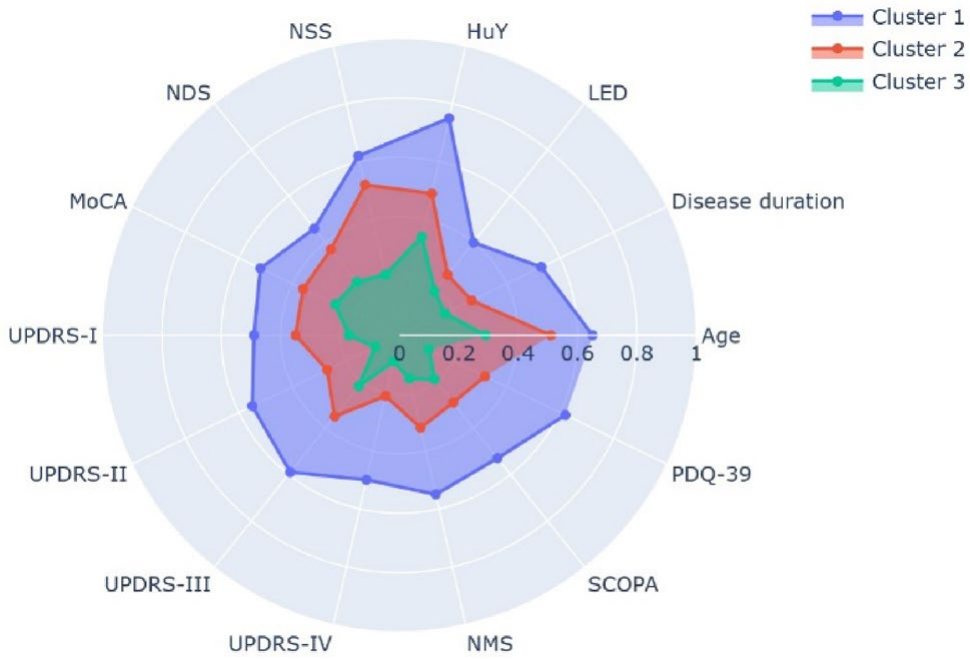
The radar chart illustrates the variations in the 14 features among the three subtypes. All values for the 14 features are standardized to a range from zero to one. As MoCA values and the other 13 variables are measured in opposite directions, with higher MoCA values indicating positive cognitive conditions and higher values for the other 13 variables corresponding to worse disease manifestations. To present the subtypes in a consistent manner within this radar chart, the original MoCA values were replaced by the new values obtained by subtracting the original data from the maximal MoCA value of 30. Subtype III (here illustrated as Cluster 3 in green) can be observed with the lowest values in all features compared to subtype I (here illustrated as Cluster 1 in blue) and subtype II (here illustrated as Cluster 2 in red)

The cluster analysis, as depicted in Figs. 3a, 4, and 5, revealed the existence of three distinct subtypes. The descriptive statistics of each subtype, as well as the results of the ANOVA and post hoc analysis between the subtypes, are presented in Tables 1 and 2.

**Table 1:** Descriptive statistics of the subtypes

Subtype	I (*N* = 40)					II (*N* = 49)					III (*N* = 25)				
	Mean	SD	Median	Min	Max	Mean	SD	Median	Min	Max	Mean	SD	Median	Min	Max
*Demographics*															
Age (year)	76.40	7.68	78	59	91	70.49	9.04	71	49	86	61.04	7.86	58	50	79
Disease duration	12.20	4.96	12	3	22	6.65	3.68	6	1	17	4.56	3.16	3	1	13
*PNP scores*															
NSS	6.22	2.46	7	0	10	5.22	2.74	5	0	10	2.08	2.53	0	0	7
NDS	4.59	2.59	4	0	10	3.65	2.53	3.69^a^	0	10	2.31	2.13	2	0	8
*Motor symptoms*															
UPDRS-Part II	22.37	8.44	22	7	41	11.03	5.07	11	3	22	3.72	2.54	3	0	9
UPDRS-Part III	42.40	12.18	40	22	70	26.58	11.02	26	10	57	17.52	8.67	16	3	41
UPDRS-Part IV	7.10	3.77	8	0	14	2.88	3.30	2	0	14	1.28	1.57	0	0	4
H&Y	3.26	0.66	3	2	4	2.46	0.58	2.5	1	4	2.02	0.53	2	1	3
*Non-motor symptoms*															
NMSQuest	14.26	4.70	14	5	25	8.79	3.23	9.76^a^	2	16	4.48	2.37	4	1	10
SCOPA-AUT	21.03	7.57	20	8	40	11.57	4.85	12	0	21	7.52	4.27	7	2	20
UPDRS-Part I	16.30	5.71	15.50^a^	7	33	11.48	4.98	11.91^a^	2	25	5.72	3.08	5	0	11
PDQ-39	69.36	20.59	62	40	111	35.98	18.63	37	1	77	13.20	8.87	10	3	38
*Cognitive function*															
MoCA	20.12	3.94	21	11	28	23.20	3.38	23	16	30	25.40	2.84	26	20	29
*Other*															
LED	895.83	402.28	791.25^a^	300	2250	584.48	346.76	515	0	1582.50^a^	421.42	361.08	310	0	1197

^a^Median values are presented with two decimal digits as the missing values were imputed using mean value

**Table 2:** ANOVA and post hoc analysis between the three subtypes

	Total sample	Subtype I	Subtype II	Subtype III	Unadjusted	Post hoc	Adjusted^a^ ANCOVA
	(*N* = 114)	(*N* = 40)	(*N* = 49)	(*N* = 25)	*P* value	*P* value	*P* value
*Sex, number (percentage)*							
Male	66 (57.9%)	21 (52.5%)	27 (55.1%)	18 (72%)	0.2867	–	–
Female	48 (42.1%)	19 (47.5%)	22 (44.9%)	7 (28%)		–	–
*Demographics, mean (SD)*							
Age (year)	70.49 (10.02)	76.40 (7.68)	70.49 (9.04)	61.04 (7.86)	< 0.0001	All comparisons	–
Disease duration	8.14 (5.10)	12.20 (4.96)	6.65 (3.68)	4.56 (3.16)	< 0.0001	All comparisons	–
*PNP scores, mean (SD)*							
NSS	4.88 (3.01)	6.22 (2.46)	5.22 (2.74)	2.08 (2.53)	< 0.0001	III versus rest	< 0.0001
NDS	3.69 (2.59)	4.59 (2.59)	3.65 (2.53)	2.31 (2.13)	0.0027	I versus III	0.6098
*Motor symptoms, mean (SD)*							
UPDRS-Part II	13.41 (9.41)	22.37 (8.44)	11.03 (5.07)	3.72 (2.54)	< 0.0001	All comparisons	< 0.0001
UPDRS-Part III	30.15 (14.59)	42.40 (12.18)	26.58 (11.02)	17.52 (8.67)	< 0.0001	All comparisons	< 0.0001
UPDRS-Part IV	4.01 (3.95)	7.10 (3.77)	2.88 (3.30)	1.28 (1.57)	< 0.0001	I versus rest	< 0.0001
H&Y	2.65 (0.77)	3.26 (0.66)	2.46 (0.58)	2.02 (0.53)	< 0.0001	All comparisons	0.0001
*Non-motor symptoms, mean (SD)*							
NMSQuest	9.76 (5.20)	14.26 (4.70)	8.79 (3.23)	4.48 (2.37)	< 0.0001	All comparisons	< 0.0001
SCOPA-AUT	14.00 (7.94)	21.03 (7.57)	11.57 (4.85)	7.52 (4.27)	< 0.0001	All comparisons	< 0.0001
UPDRS-Part I	11.91 (6.26)	16.30 (5.71)	11.48 (4.98)	5.72 (3.08)	< 0.0001	All comparisons	< 0.0001
PDQ-39	42.70 (27.82)	69.36 (20.59)	35.98 (18.63)	13.20 (8.87)	< 0.0001	All comparisons	< 0.0001
*Cognitive function, mean (SD)*							
MocA	22.60 (4.00)	20.12 (3.94)	23.20 (3.38)	25.40 (2.84)	< 0.0001	All comparisons	0.0027
*Other, mean (SD)*							
LED	657.97 (411.55)	895.83 (402.28)	584.48 (346.76)	421.42 (361.08)	< 0.0001	I versus rest	0.2268

Chi-square tests were performed for categorical data, and for continuous features, ANOVA (analysis of variance) with Kruskal–Wallis *H* test and post hoc analysis with Dunn’s test with Holm adjustment were performed

^a^ANCOVA (analysis of covariance) was performed to adjust between-clusters comparisons for age and disease duration as potential covariates

Subtype I included 40 patients (21 males, 19 females, mean age 76.40 ± 7.68 years). Those patients had the highest PDQ-39 scores (69.36 ± 20.59), the most severe motor and non-motor symptoms, which are demonstrated by the highest MDS-UPDRS Part II scores (22.37 ± 8.44) and NMSQuest scores (14.26 ± 4.70). Subtype III was the youngest group with 25 patients (18 males, 7 females, mean age 61.04 ± 7.86 years). Patients in this group had the least affected motor and non-motor impairment in all domains. They exhibited the mildest cognitive impairment as demonstrated by the highest MoCA score (25.40 ± 2.84). Forty-nine patients belonged to subtype II (27 males, 22 females, mean age 70.49 ± 9.04 years), presenting an intermediate score in both motor and non-motor components.

The data presented in Table 2 indicate a substantial clinical differentiation among the subtypes with respect to the mean values of various variables, including age, disease duration, scores on the PNP (NSS and NDS), MDS-UPDRS Part I, II, and III, H&Y, NMSQuest, SCOPA-AUT, PDQ-39, and MoCA. After adjusting for the confounders of age and disease duration using ANCOVA, the subtypes continue to exhibit statistically significant differences (*P* < 0.01) in relation to all of the previously mentioned variables, with the exception of NDS.

The variations in clinical characteristics between the mean values of each cluster are illustrated using a radar chart (Fig. 5). Individuals belonging to subtype III were found to be younger and exhibited the least severe motor and non-motor symptoms, consistent with the shortest disease duration. Conversely, individuals belonging to subtype I displayed the most severe motor and non-motor manifestations, along with the most impaired cognitive function and older ages. Individuals belonging to subtype II were characterized by intermediate values between subtype I and III. As a result, subtype I can be classified as a late-onset severe type, subtype III as an early onset mild type, and subtype II as an intermediate type.

## Discussion

The clinical variability between PD patients suggests the existence of subtypes of the disease. Identification of subtypes is important, since a focus on homogeneous groups may enhance the chance of success of research on mechanisms of disease and may also lead to tailored treatment strategies (van Rooden et al. 2010). Defining subtypes of PD is, therefore, needed to better understand underlying mechanisms and predict disease course (Fereshtehnejad et al. 2015). In this study, the authors developed a data-driven subtyping method for 114 patients with idiopathic PD. A wide spectrum of motor and non-motor variables was included in clustering analysis and three unique subtypes emerged:

- Subtype I, comprised 40 patients, was characterized as late-onset severe type.
- Subtype II, comprised 49 patients, was identified as an intermediate type.
- Subtype III, comprised 25 patients, was characterized as the early onset mild type.

The current clustering analysis provides evidence that the PDQ-39, NMSQuest, and the MDS-UPDRS Part II are the most crucial variables in differentiating patients with PD. A strong correlation was observed between the PDQ-39 and the MDS-UPDRS Part II (Spearman Rs = 0.796; *P* < 0.001) as demonstrated in Fig. S4, which serves as an indicator of the alignment between the patient-reported quality-of-life measurement and the motor experience of daily life. Additionally, a significant correlation was found between PDQ-39 and NMSQuest (Spearman Rs = 0.699; *P* < 0.001), as illustrated in Fig. S4. This is an indicator for the agreement between the patient-reported quality-of-life measurement and the non-motor symptoms. Previous research has already demonstrated that non-motor symptoms, as measured by the Non-Motor Symptoms Scale (NMSS), have the most significant impact on the health-related quality of life (Hr-QoL) of PD patients (Li et al. 2010). The current study confirms this notion by demonstrating a strong correlation between the PDQ-39 and the NMSQuest, which measure the patient-reported quality of life and the non-motor symptoms, respectively.

In the current analysis, PDQ-39 had the highest quality of representation of all the 14 variables and the largest loading score in the current PCA analysis. PDQ-39 is the most widely used patient-reported rating scale in PD (Hagell and Nygren 2007) and a reliable evaluation of PD on both motor and non-motor aspects. PDQ-39 was also found to have the largest effect size to measure QoL (quality of life) by PD in the meta-analysis (Zhao et al. 2021).

While PDQ-39 is widely used to assess QoL in PD patients, it has certain limitations. Several studies have demonstrated the importance of non-physical factors, such as education, disease acceptance, and financial background in determining the quality of life in PD patients. Jenkinson et al. (1997) found that higher levels of education were associated with better overall quality of life in PD patients. The study conducted by Cubo et al. (2002) emphasized the role of education and psychological factors in determining the QoL of PD patients, particularly in emotional and social domains. Schrag et al. (2000) reported that disease acceptance was a crucial factor in determining the QoL in PD patients, as is financial background. Notably, financial stressors can impede patients' access to medical care, medications, and resources needed to manage their condition. Overall, healthcare professionals should consider these factors to provide targeted care and improve patients' experience.

Like PDQ-39, NMSQuest made a crucial contribution to form the three subtypes. Several studies have demonstrated that non-motor symptoms are important to define features of PD subtypes (Marras 2015; Zella et al. 2019). In a previous cluster analysis of PD, a separate non-motor dominant subtype was described (Erro et al. 2013). Another study (Fereshtehnejad et al. 2015) found that the best cluster solution was based on non-motor features. The NMSQuest has been developed as a patient-reported instrument to evaluate a broad spectrum of non-motor symptoms (Chaudhuri et al. 2006). In the current analysis NMSQuest scores of the three subtypes (14.26 ± 4.70, 8.79 ± 3.23, and 4.48 ± 2.37 for subtype I, II, and III, respectively) are consistent with the cut-off points of NMSQuest grading system proposed by (Chaudhuri et al. 2015): very Severe: > 14; severe: 10–13, moderate: 6–9, and mild: 1–5. Since NMSQuest was not developed for measuring the severity of symptoms (Chaudhuri et al. 2006), the Movement Disorder Society Non-Motor Rating Scale (MDS-NMS), which was introduced in 2019, utilizes a novel approach to evaluate non-motor symptom severity by computing a total score through the multiplication of symptom severity and its frequency. This approach offers a more precise method of assessing non-motor symptoms, as it considers both the intensity and the frequency of the symptoms.

The MDS-UPDRS Part II (motor experiences of daily living) captures the impact of PD on daily function and it was included in the analysis as an important variable by most of the previous cluster analysis. The self-rated MDS-UPDRS Part II proved to be useful for assessing disability in PD and showed a better performance than other rater-based, generic or specific, scales to assess disability in PD (Rodriguez-Blazquez et al. 2013; Rodríguez-Blázquez et al. 2017). As a remarkable variable in the present cluster analysis, a strong correlation was found between MDS-UPDRS Part II and PDQ-39 (Spearman Rs = 0.796; *P* < 0.001), which is consistent with a previous study (Skorvanek et al. 2018): health-Related Quality of Life (HRQoL), which was measured by PDQ-8 (a shortened version PDQ-39), was found significantly related to MDS-UPDRS Part II (ADLs) and Part I (NMS). A previous study found a strong correlation between the scores on the MDS-UPDRS Part II and the duration of the disease in 888 patients with idiopathic PD. The results of this study suggest that a single measurement of UPDRS II scores may be a good marker of disease progression than other scores on the MDS-UPDRS scale (Harrison et al. 2009).

To minimize the influence of comorbidities that may confound the interpretation of neuropathy scores in the current cluster analysis, the authors implemented stringent exclusion criteria that excluded individuals with a diagnosis of diabetes mellitus or alcohol dependence disorder, as well as other conditions that may cause neuropathy. Furthermore, an earlier study of the same authors indicated that there was no significant correlation between LED and tibial nerve compound muscle action potential (cMAP) (Kühn et al. 2020). This finding reinforces the validity and reliability of the polyneuropathy scores applied here and allows the authors to utilize them with greater confidence in the current cluster analysis, thereby reducing the risk of potential bias. The polyneuropathy scores (NSS and NDS) have the lowest quality of representation values in the cluster analysis and therefore the least contribution to distinguish between subtypes. The patient-reported polyneuropathy symptoms correlate weakly with motor- and non-motor symptoms: NSS and MDS-UPDRS Part II (Spearman Rs = 0.293; *P* = 0.001), and NSS and NMS (Spearman Rs = 0.368; *P* < 0.001). No correlation could be observed between NDS and MDR-UPDRS Part II (Spearman Rs = 0.093; *P* = 0.328) and between NDS and NMS (Spearman Rs = 0.116; *P* = 0.221). Despite the high prevalence of polyneuropathy among patients with PD being reported in the previous studies (Crespo-Burillo et al. 2016; Kühn et al. 2020), it was not a major determinant of patient subtypes in the current cluster analysis. Additionally, even after adjusting for factors such as age and disease duration, no significant differences in the Neuropathy Disability Score (NDS) were observed between the clusters.

The PDQ-39, NMSQuest, and MDS-UPDRS Part II are self-reporting questionnaires that are easy to apply and do not require specialized training or equipment, making them accessible to clinicians and researchers. Furthermore, the use of self-reported questionnaires allows patients to provide insight into their own experiences with PD symptoms, potentially leading to more accurate assessments of their symptoms. They are essential tools in differentiating subtypes of Parkinson's disease (PD) and evaluating the quality of life of PD patients. These questionnaires provide a comprehensive assessment of various aspects of the disease, including patient-reported quality of life, non-motor symptoms, and motor function, respectively. They are easily accessible way to gather important information about PD patients, which can aid in diagnosis, treatment planning, and monitoring of disease progression.

### Comparison with other cluster analysis methodologies

The three-cluster solution found in the current study aligns with the results of previous research that used either *k*-means or hierarchical clustering as the method of grouping. The clustering of domains in patients with PD shows a consistent pattern, indicating the validity and reliability of these clustering techniques. Post et al. (2008) identified three clusters, including a group with younger onset, an intermediate group with older onset, and an oldest onset group. Three subtypes were also defined by Fereshtehnejad et al. (2015) as mainly motor/slow, diffuse/malignant, and intermediate progression. Another study by Fereshtehnejad et al. (2017) conducted a cluster analysis of 421 PD patients from the PPMI Database and identified three PD subtypes: mild motor-predominant, diffuse malignant, and intermediate subtype. Based on clinical and biomarker data, Zhang et al. (2019) also described three PD subtypes: Subtype I (Mild Baseline, Moderate Motor Progression), Subtype II (Moderate Baseline, Mild Progression), and Subtype III (Severe Baseline, Rapid Progression). Krishnagopal et al. (2020) applied Trajectory Profile Clustering (TPC) and found three distinct clusters: mixed subtype, mild subtype, and severe subtype.

The present clustering analysis demonstrated that PDQ-39, NMSQuest, and the MDS-UPDRS Part II were the crucial variables to differentiate the patients. Other variables like MDS-UPDRS Part I, SCOPA, MDS-UPDRS Part III, H&Y and disease duration contributed substantially to the formation of the clusters (Fig. 3b). A previous PD clustering study (Fereshtehnejad et al. 2015) came to a similar conclusion: the most informative variables in generating clusters were identified including UPDRS Part II, UPDRS Part III, REM sleep behavior disorder (RBD), mild cognitive impairment (MCI), Orthostatic hypotension, depression, and anxiety.

Age is considered a main risk factor for developing PD (Elbaz et al. 2016). The progression of PD is slower in early onset PD (Ferguson et al. 2016), on the contrary, older age at onset was associated with a more severe motor and non-motor phenotype (Pagano et al. 2016). A recent study compared 24 PD cluster analysis research between the years of 1990–2021 and a series of limited age ranges were discovered among those cluster solutions: the smallest difference in minimum and maximum patient cluster ages was 3.7 years, which was among three clusters. While the largest difference between patient cluster ages was 12.4 years (Reijnders et al. 2009; Hendricks and Khasawneh 2021). The current cluster subtypes had an age range of 15.4 years between the early onset mild type and late-onset severe type.

In previous studies on PD clusters, the use of silhouette scores was not reported (Hendricks and Khasawneh 2021). The average silhouette score is a commonly used method to determine the optimal number of clusters prior to analysis and evaluate the results of clustering. The current study incorporated both silhouette score and Calinski–Harabasz score elbow to validate the cluster solution.

### Limitations

Limitations of the study include the exclusive use of data from clinic-recruited PD patients, resulting in a small sample size of 114 patients, which may have impacted the machine learning algorithm's efficacy and the ability of the analysis to capture the full data variability. Relying on clinic-recruited patients may also limit diversity and generalisability. These limitations should be considered when interpreting and applying the findings to a larger population. Future studies with larger and more diverse patient populations are needed to validate the findings and improve generalisability. Furthermore, longitudinal data are critical for an understanding of the stability of proposed subtypes (Mestre et al. 2021). In this regard, a longitudinal follow-up study is to be continued and will be carried out at the Department of Neurology, St. Josef-Hospital, at the Ruhr University in Bochum (Germany), to compare the prognosis and progression rate between the identified subtypes. The analysis conducted in this study did not incorporate the use of biomarkers and imaging techniques, which could have provided additional insights into the subtypes of PD. Despite this limitation, the clustering analysis performed in this study provides a useful starting point for understanding PD subtypes. However, it is important to note that the subtyping of PD presented in this study must be validated through further research in clinical practice to establish its reliability and validity.

## Conclusions

Three distinct PD subtypes were identified using *k*-means++ cluster analysis: late-onset severe type, intermediate type, and early onset mild type. Through PCA and between-cluster comparison, self-reporting questionnaires, such as the PDQ-39, NMSQuest, and the MDS-UPDRS Part II, were found to be the crucial factors to differentiate PD subtypes and evaluate the PD heterogeneity. They are easy to administer, accessible, and provide subjective insight into patients' experiences with PD symptoms, enhancing symptom assessments' accuracy. These questionnaires are valuable for identifying and classifying PD subtypes, enhancing disease understanding, and informing clinical practice and patient care. By identifying a statistical relationship, this research provides a solid foundation for defining different subtypes of PD, making the clustering process highly differentiated and effective. Finally, future works should aim at analyzing the longitudinal trend of progression between the different subtypes in the sense of a follow-up of years, to identify patients and patient groups with different rates of progression and how this relates to their clinical characteristics in the early years of the disease.

## Supplementary Information

Below is the link to the electronic supplementary material.

Supplementary file 1 (DOCX 914 KB)

## Data Availability

The datasets generated during and/or analyzed during the current study are available from the corresponding author on reasonable request.

## Abbreviations

H&Y: Hoehn and Yahr Stage
LED: Levodopa dosage
MDS-UPDRS: Movement Disorder Society-Unified Parkinson’s Disease Rating Scale
MoCA: Montreal Cognitive Assessment
NDS: Modified Neuropathy Disability Score
NMS or NMSQuest: Non-motor Symptom Questionnaire
NSS: Modified Neuropathy Symptom Score
PCA: Principal Component Analysis
PDQ-39: Parkinson’s Disease Questionnaire
SCOPA-AUT: Scales for Outcomes in Parkinson’s Disease-Autonomic
