# Supplementary material for: Data-driven subtyping of Parkinson’s disease: comparison of current methodologies and application to the Bochum PNS cohort
Source: J Neural Transm (Vienna). 2023 Mar 31;130(6):763–76. doi: 10.1007/s00702-023-02627-4 (PMC10199871; doi:10.1007/s00702-023-02627-4)
Supplement: Supplementary file 1 — (DOCX 914 KB) [file 702_2023_2627_MOESM1_ESM.docx]

**Electronic Supplementary Material**

**
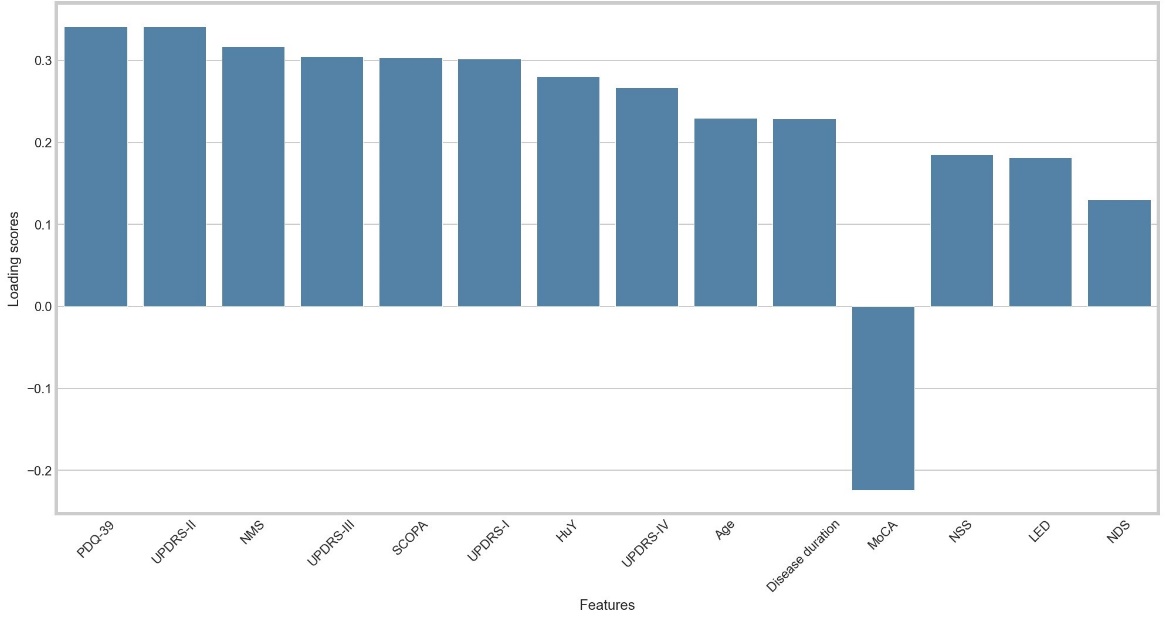
**

**
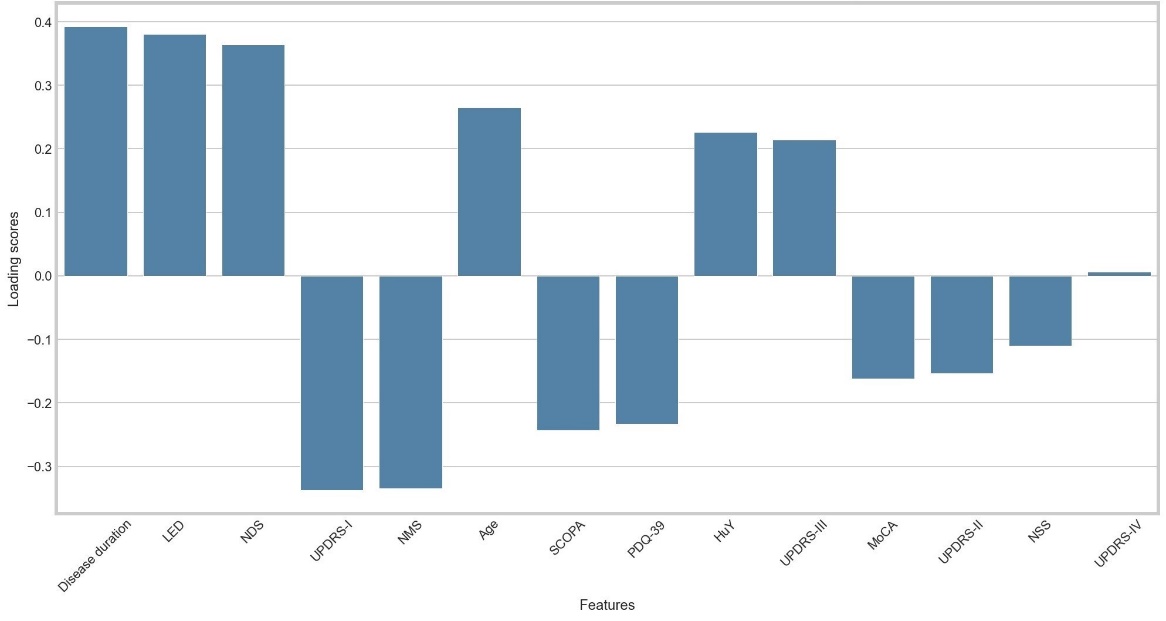
**

**Fig. S1** PCA loading scores of the fourteen features for the first principal component (top) and the second principal component (bottom), the features are sorted by the absolute values


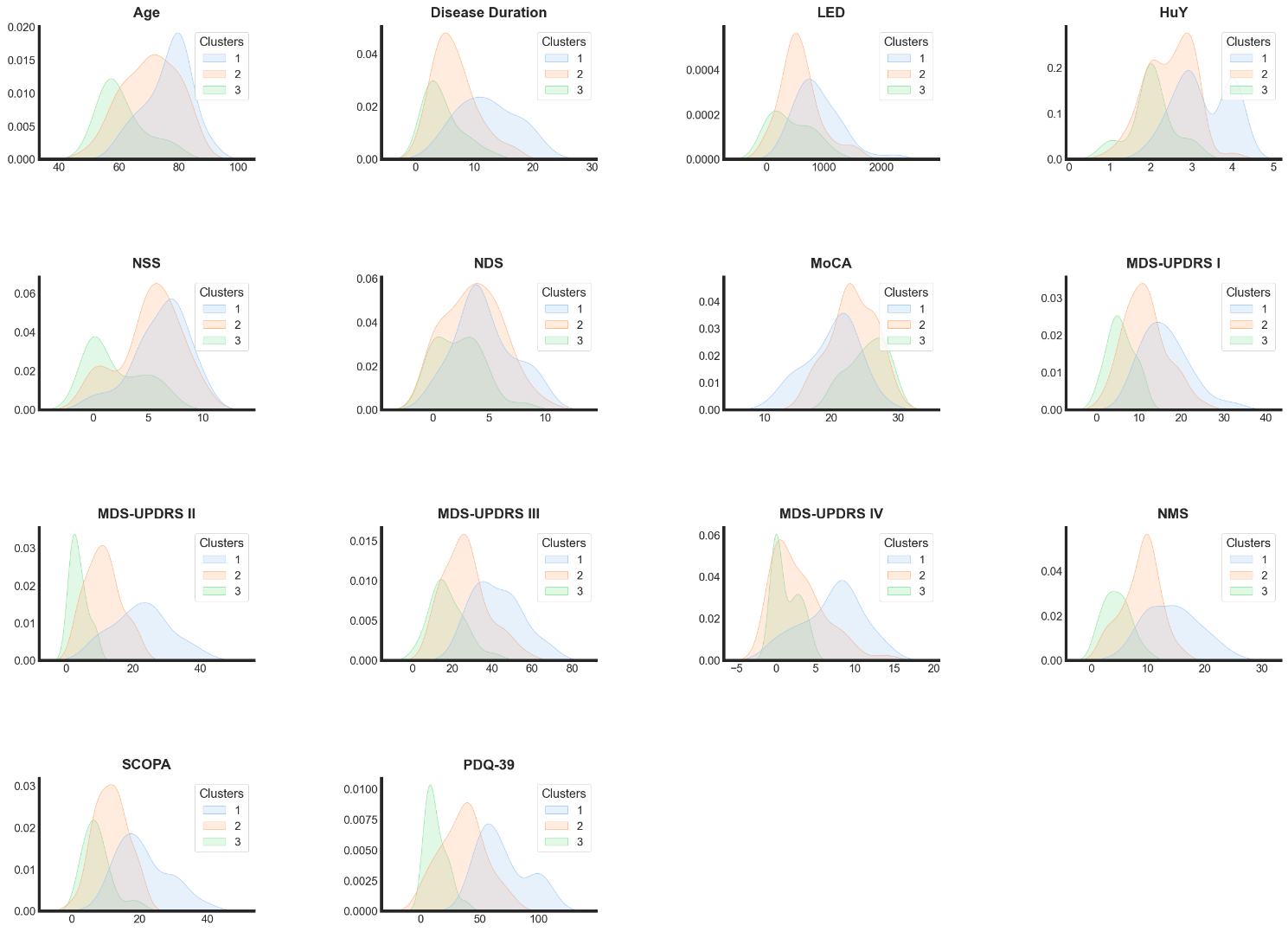


**Fig. S2** Distribution of the fourteen features by clusters


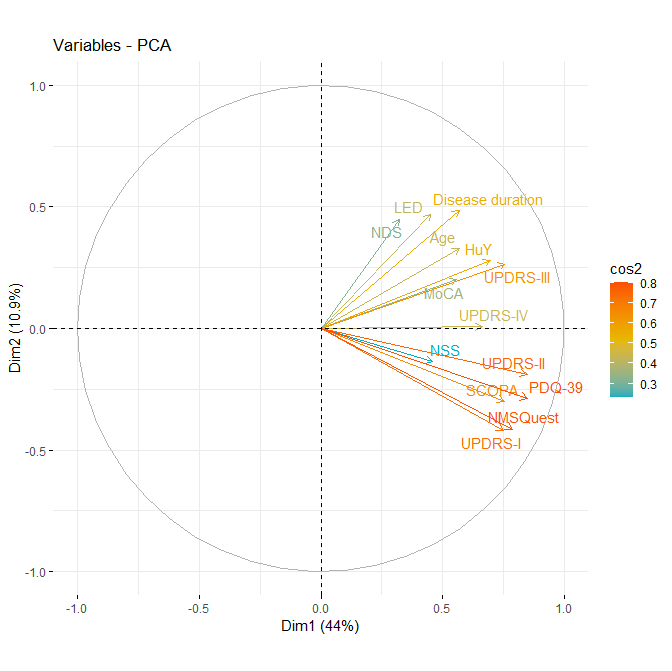


**Fig. S3** Correlation circle of the fourteen features. As MoCA values and the other thirteen variables are measured in opposite directions, with higher MoCA values indicating positive cognitive conditions and higher values for the other thirteen variables corresponding to worse disease manifestations. In order to present the subtypes in a consistent manner, the original MoCA values were replaced by the new values obtained by subtracting the original data from the maximal MoCA value of 30


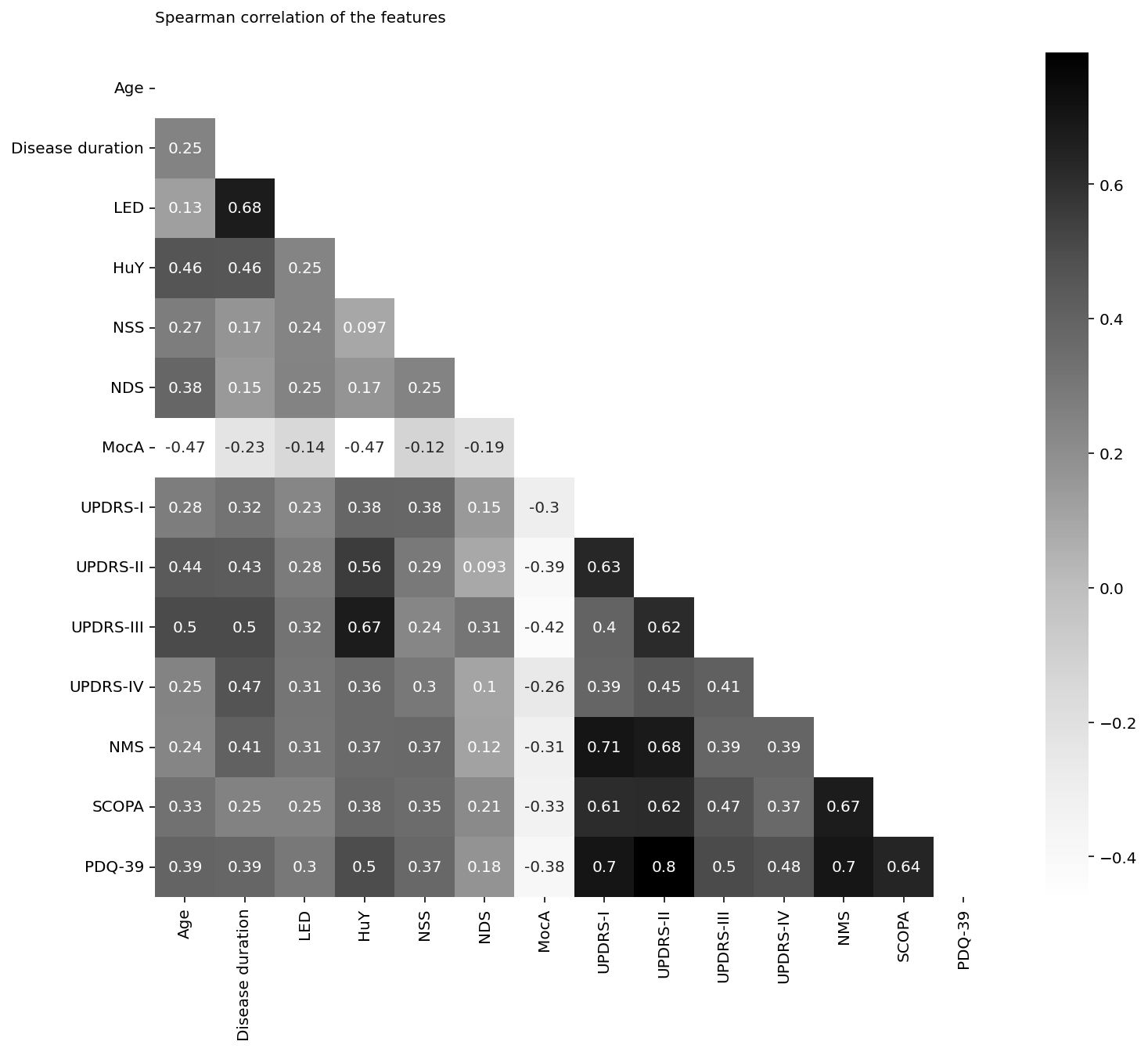


**Fig. S4** Spearman correlation of the fourteen features
